# The Role of Vasodilator-stimulated Phosphoproteins in the Development of Malignant Tumors

**DOI:** 10.2174/0115680096262439231023110106

**Published:** 2024-03-14

**Authors:** Jiandong Gui, Hangsheng Zhou, Hongyuan Wan, Dongjie Yang, Qing Liu, Lijie Zhu, Yuanyuan Mi

**Affiliations:** 1 Wuxi School of Medicine, Jiangnan University, 1800 Lihudadao, Wuxi, 214122, Jiangsu Province, China;; 2 Department of Urology, Affiliated Hospital of Jiangnan University, 1000 Hefeng Road, Wuxi, 214122, Jiangsu Province, China;; 3 Huadong Sanatorium, 67 Dajishan, Wuxi 214122, Jiangsu Province, China

**Keywords:** Malignant tumors, vasodilator-stimulated phosphoprotein, invasion, metastasis, biomarker, function

## Abstract

Vasodilator-stimulated phosphoprotein (VASP) is an actin-binding protein that includes three structural domains: Enabled/VASP homolog1 (EVH1), EVH2, and proline-rich (PRR). VASP plays an important role in various cellular behaviors related to cytoskeletal regulation. More importantly, VASP plays a key role in the progression of several malignant tumors and is associated with malignant cell proliferation, invasion, and metastasis. Here, we have summarized current studies on the impact of VASP on the development of several malignant tumors and their mechanisms. This study provides a new theoretical basis for clinical molecular diagnosis and molecular targeted therapy.

## 1 INTRODUCTION

Cancer remains a major health problem, with 1,918,030 new cancer cases and 609,360 cancer deaths worldwide in 2022 [1]. The elevated mortality rate is because most cancers are not easily detected in their early stages, and at an advanced stage, they are difficult to cure. Management of cancer at early diagnosis and effective treatment after late detection are crucial for reducing disease burden. With China's aging population and changing risk factors, the incidence of new cases and mortality related to cancer is higher than that in any other country [2]. Some pioneers have conducted many outstanding studies on malignant tumor therapies. Ye *et al.* [3] found that nanoplatelets (platelet membranes on the surface of nanoparticles) can achieve therapeutic effects by destroying circulating tumor cells in breast cancer anti-metastatic therapy. Chin *et al.* [4] developed cluster-structured nanoparticles (CNPs) and found that CNPs can be used in combination with photodynamic therapy to induce ferroptosis to achieve therapeutic effects, and this new type of therapy has the potential to promote immunotherapy. Ye *et al.* [5] combined Golgi apparatus-PD-L1 (programmed death-ligand 1)-/-exosome hybrid membrane coated nanoparticles (GENPs) and anti-PD-L1 immunotherapy through the sprayable *in situ* hydrogel, prolonging survival periods in mice models with incomplete metastatic melanoma resection. Therefore, the development of novel cancer therapies is essential. In the process of reading a large amount of literature, we have found a promising new target for the treatment of malignant tumors.

**Fig. (1):**
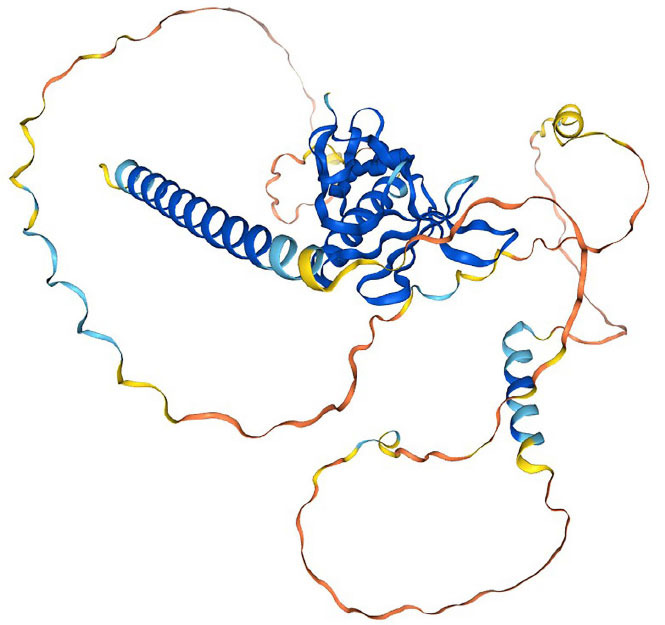
Structure prediction from Alphafold project (alphafold.com).

The vertebrate Ena/VASP (enabled/vasodilator-stimulated phosphoprotein) family is a class of actin-binding proteins that includes three members: mammalian Ena (Mena), VASP, and Ena/VASP-like proteins [6]. VASP, which was first identified in platelets in 1990 (Fig. **1**) [7], is widely distributed in the body and is abundant in the lungs, stomach, large intestine, smooth muscles, brain, heart, kidneys, and small intestine. In these tissues and organs, VASP is expressed mostly in platelets, fibroblasts, vascular endothelial cells, smooth muscle cells, and to varying degrees in the cells of other tissues [8]. Previous studies have demonstrated the significance of VASP in the development of filopodia in mammalian cells, as it primarily localizes to sites of active actin assembly [9-11]. In this review, we have summarized the fundamental features of VASP and its modulation in various malignant tumors.

## 2 STRUCTURAL CHARACTERISTICS OF VASP

VASP is a 47 kDa cell membrane-associated protein with three phosphorylation sites: Ser157, Ser239, and Thr278. Ser157 is the site of action of 3',5'-cyclic adenosine monophosphate (cAMP)-dependent protein kinase A (PKA), Ser239 is the site of action of 3′,5′-cyclic guanosine monophosphate (cGMP)-dependent protein kinase G (PKG), and Thr278 is the site of action of adenosine monophosphate (AMP) kinase [12]. Other studies have reported that all three sites are phosphorylated by PKA and PKG [13, 14]. However, phosphorylation activities at these three sites differ [15].

**Fig. (2):**
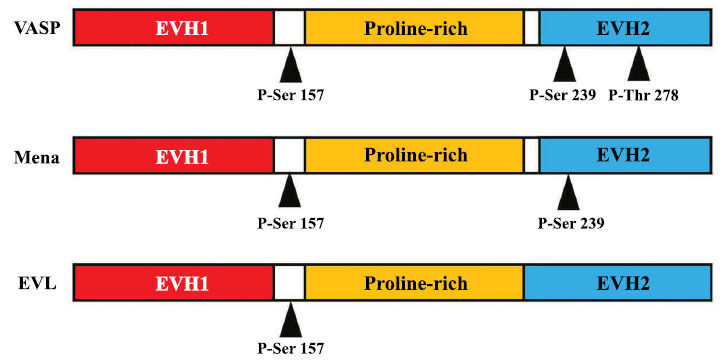
The vertebrate Ena/VASP family phosphorylation. Three members of the mammalian Ena/VASP family are depicted in the cartoon (not to scale). VASP has three phosphorylation sites (Ser-157, Ser-239, and Thr-278), while Mena contains the first two, and EVL has only the first site [77-79].

The Ena/VASP protein family possesses three structural regions: the amino-terminal EVH1 (Ena/VASP homolog1) domain, central proline-rich domain (PRR), and carboxy-terminal EVH2 domain (Fig. **2**) [12]. The EVH1 domain can bind to proteins in the proline-rich region to form a polyproline II helix (PPII helix) that performs various functions [16]. The proline-rich EVH1 ligand is also a proline-rich protein that binds directly to the EVH1 region of VASP and Ras, thereby activating the Ras signaling pathway for cytoskeletal rearrangement during cell migration and extension [17]. The PRR domain binds to the Src homology 3 (SH3) domain, WW domain (a small residue module of 38-40 amino acids involved in the binding of proline-rich sequences), and another important actin regulatory protein, profilin. One of the three phosphorylation sites of VASP Ser157 is located in this region. The EVH2 domain consists of 160-190 amino acids and can be further divided into three parts from the amino terminus to the carboxyl terminus as follows: A (225-245), B (259-278), and C (343-377). The phosphorylation site of VASP Ser239 is in region A. VASP binds to actin filaments in region B, which contains the phosphorylation site Thr278, and VASP forms a tetramer in region C [18].

## 3 MOLECULAR FUNCTIONS OF VASP

VASP performs multiple functions both *in vivo* and in cell culture systems. Fibroblast motility is regulated by Ena/VASP proteins [19, 20]. Actin cytoskeletal remodeling and T-cell signaling are crucially linked through the involvement of VASP [21]. Effective Jurkat T-cell polarization and phagocytosis require properly localized Ena/VASP proteins and actin-related protein (Arp) 2/3, respectively [22]. Ena/VASP proteins play key roles in neuronal migration [23].

Several studies have identified three possible molecular mechanisms underlying Ena/VASP function: anti-capping, anti-branching, and profilin recruitment. VASP reduces the branching of actin filaments and facilitates the continuous lengthening of actin [20]. Overexpression of VASP at the pseudopod site antagonizes the activity of the capping protein, resulting in the formation of elongated actin filaments with fewer branches, which increases the protrusion rate of the lamellar pseudopod. However, the ability of these filaments to resist membrane tension is weak, and thus decreases cell movement. In contrast, the absence of VASP results in a network of highly bifurcated short actin filaments with high resistance to tension, leading to faster cell migration. Ena/VASP proteins can also inhibit Arp2/3-mediated branching of actin filaments or reduce branching stability [24]. The Listeria monocytogenes ActA protein stimulates the Arp2/3 complex *via* both VASP-dependent and -independent mechanisms, resulting in distinct populations of actin filaments in L. monocytogenes comet tails. VASP promotes actin filament nucleation and regulates actin filament structure. This underscores the central role of VASP in the movement of actin. Profilin-actin is recruited by the last poly-Pro fragment of VASP and binds to the G-actin binding (GAB) domains of poly-Pro and VASP. It is proposed that the actin monomer bound to the GAB structural domain is added to the barbed end of the growing filament at the same time as profilin is released.

## 4 RELATIONSHIP BETWEEN VASP AND MALIGNANT TUMORS

The role of the cytoskeleton in shaping oncogenic cells has been highlighted in various studies [25, 26]. As a regulatory protein for the cytoskeleton, VASP may be involved in the development of malignant cells. Liu *et al.* [27] used a random pure knockdown technique to downregulate VASP expression in NIH 3T3 fibroblasts, which in turn caused persistent cell division and potential oncogenicity. Surprisingly, VASP overexpression also led to NIH 3T3 tumor formation, suggesting that normal cell growth may require the control of VASP protein expression within appropriate limits.

### 4.1 VASP and Breast Cancer

Han *et al.* [28] suggested VASP to be overexpressed in MDA-MB-231, a highly motile breast cancer (BC) cell line, compared to MCF-7 cells, and that high levels of VASP expression enhanced the aggressive migration of human BC cells through the Ras-related C3 botulinum toxin substrate 1 (Rac1) pathway. This study indicated a strong association between VASP expression and BC cell development. Su *et al.* [29] identified a novel tumor necrosis factor-alpha (TNF-α)/hypoxia-inducible factor-1alpha (HIF-1α)/VASP axis, in which HIF-1α acts downstream of TNF-α to inhibit VASP expression and regulates adhesion and proliferation of BC cells. Similar results have been reported previously. Changchun *et al.* [30] suggested that IL-17 effectively enhanced the TNF-α-induced increase in HIF-1α and could reduce the adhesion of MDA-MB-231 cells by inhibiting the expression of VASP. Su *et al.* [31] showed cell migration in basal-like BC to be inhibited by the binding of the tumor suppressor berberine to VASP. This suggests the possibility of developing new targeted drugs for BC treatment. Wang *et al.* [32] elucidated the morphological mechanism by which VASP knockdown changes the ultrastructure of MCF-7 cells. This study contributes greatly to our understanding of the mechanism of VASP in BC. Gkretsi *et al.* [33] showed that silencing of VASP in MDA-MB-231 cells inhibited tumor spheroid invasion by downregulating the expression of migfilin, β-linked protein, and urokinase-fibrinogen activator, and that high VASP expression was associated with poor remission-free survival in lymph node-positive BC patients. This suggests that VASP is a potential novel biomarker for BC metastasis. Subsequent studies have shown that VASP regulates BC cells through several different signaling pathways. Hu *et al.* [34] showed that VASP, a key oncogene, interacts with other molecules to form a cAMP-responsive element binding protein 1 (CREB1)/Lin28/miR-638/VASP network that promotes the proliferation and migration of BC. Li *et al.* [35] revealed for the first time that VASP is located in the nucleus of BC cells and illustrated that VASP mutually promotes activation of the canonical Wnt/β-catenin pathway to form a malignant positive feedback loop, thereby promoting the proliferation and migration of BC cells. Epithelial mesenchymal transition (EMT) is an important feature of cancer, and tumor invasion and early metastasis are strongly correlated with EMT [36]. The study by Zhang *et al.* [37] linked VASP, EMT, and BC, and explained the mechanisms involved. It was shown that integrin α3 interacts with VASP to regulate stem cell properties, EMT, and phosphatidylinositol 3-kinase/protein kinase B (PI3K/AKT) pathways in BC cells. In a recent study, Barone *et al.* [38] designed selective small-molecule inhibitors that impaired the invasion and extravasation of BC cells by targeting Ena/VASP interaction with EVH1. This indicates that targeted VASP has great potential for the treatment of BC. VASP overexpression contributes to increased tumor growth and progression. VASP is also associated with increased invasion and metastasis, which can hinder the treatment of BC. Targeting VASP using drugs may prevent cells from becoming cancerous, or slow the spread of cancer. In conclusion, the role of VASP in BC is still being studied and further research is required to comprehend its function and how it can be used to treat BC. Thus far, research in this area has provided promising insights into the importance of VASP and how it can be used to fight BC.

### 4.2 VASP and Gastric Cancer

Tao *et al.* [39] determined the localization of VASP in gastric cancer (GC) cells and used SGC-7901 cells to conduct experiments. The results showed that p-VASP co-localized with α-tubulin at spindle poles and fibers during prophase, mid-phase, and late mitosis in the GC cell line SGC-7901. Gao *et al.* [40] identified and validated isobaric tags for relative and absolute quantitation (iTRAQ) coupled with liquid chromatography-mass spectrometry, and found VASP as differentially expressed in GC and adjacent normal tissues. These studies suggest that VASP phosphorylation may be linked to the development of gastric carcinogenesis. Several studies have indicated that VASP regulates the multiple biological behaviors of GC cells through various signaling pathways. Wang *et al.* [41] suggested that icariin negatively affects GC cell invasion and metastasis *via* the Rac1-dependent VASP pathway. Wang *et al.* [42] first revealed miR-610 as a new epidermal growth factor (EGF)-regulated miRNA that targets VASP in GC cells, suggesting the possibility of artificially interfering with the EGF-miR610-VASP axis to inhibit the GC development. Chen *et al.* [43] suggested that miR-4455 targets the VASP-mediated activation of the PI3K/AKT signaling pathway and inhibits VASP-mediated proliferation, migration, and invasion of GC cells. Chen *et al.* [44] suggested that betulinic acid could downregulate VASP expression by inhibiting nuclear factor-kappaB (NF-κB), thereby suppressing the GC cells' progression. Zhang *et al.* [45] showed matrine to regulate the structure, subcellular localization, expression, and phosphorylation of VASP in GC cells, thereby attenuating the adhesion and migration of cancer cells. In summary, VASP was closely linked to the development and progression of GC. Studies have shown that VASP expression is high in advanced GC and has the potential to act as an independent predictor of unfavorable patient prognosis. In summary, VASP interacts with other molecules to influence GC cell development, and this process can be reversed through manual intervention. VASP expression is elevated in GC tissues compared to normal gastric tissues, and has the potential to serve as an independent predictor of poor patient prognosis. Further studies are required to clarify whether VASP is an important biomarker for early tumor diagnosis and as a therapeutic target.

### 4.3 VASP and Lung Cancer

Dertsiz *et al.* [46] suggested that VASP is differentially expressed in normal and lung cancer (LC) tissues, and the pathological stage of cancer tissues is positively correlated with VASP, indicating that VASP may promote the differentiation of normal lung cells into cancer cells and may enhance the invasive behavior of LC. Subsequently, Liu *et al.* [47] suggested that the HIF-1α/VASP signaling pathway plays an important role in regulating TNF-α-induced inhibition of A549 cell proliferation and xenograft growth, which provides an opportunity to develop new therapeutic tools for LC. Under these conditions, VASP not only shows differences in scientific studies, but also correlates with LC at the clinical level. As the specific molecular mechanisms of VASP and LC and the regulation of proliferation, migration, invasion, and apoptosis of LC cells have not been thoroughly studied, it cannot be conclusively stated whether VASP can be applied in clinical treatment or used as a new tumor marker. Thus, thorough investigations are required in this area of study.

### 4.4 VASP and Hepatocellular Carcinoma

Liu *et al.* [48] suggested VASP as expressed more in hepatocellular carcinoma (HCC) tissues than in normal tissues, and it was reported to be positively associated with metastatic potential. The results showed that the upregulation of VASP expression can be mediated by various hypoxia-induced molecular mechanisms, such as the activation of AKT and ERK signaling by CRK-like protein (CRKL), HIF-1α, miR-204, and transforming growth factor beta (TGF-β), to promote the expression of EMT and matrix metalloproteinases (MMPs). In addition, VASP can modify the activation of signaling pathways involved in cell growth and proliferation, making it a promising target for cancer therapy. In addition to the molecular mechanisms underlying the induction of hypoxia, VASP interacts with other molecules to mediate HCC progression. Gkretsi *et al.* [49] showed that silencing the expression of migfilin in HepG2 cells led to the upregulation of actin recombination-associated proteins, such as phospho-VASP (Ser157 and Ser239), Fascin-1, and Rho-kinase-1, thereby facilitating actin polymerization and attenuating cell invasion. Dang *et al.* [50] suggested that VASP expression promoted HCC metastasis, and HOXC10 was upregulated by interleukin-1beta (IL-1β) to activate the expression of VASP. In summary, VASP can interact with a variety of HCC-related molecules to promote HCC invasion and metastasis. VASP is a novel candidate target for the diagnosis and treatment of HCC. However, the involvement of VASP in hepatocarcinogenesis and development remains relatively poorly understood, and more in-depth studies are needed to elucidate other mechanisms.

### 4.5 VASP and Colon Cancer

Ali *et al.* [51, 52] showed that disruption of VASP Ser157 phosphorylation reduces VASP function in human colon cancer (CC) cells, including their clonogenic and migratory capacity. This function was similar to the regulation of VASP Ser239 by 8-chlorophenylthio 3′,5′-cyclic guanosine monophosphate (8-CPT-cGMP). In contrast, blocking VASP Ser239 phosphorylation increases cellular clonogenicity and migration. This function is similar to that of 8-Br-cAMP-dependent regulation of VASP Ser157. Zuzga *et al.* [53] also found that rapid phosphorylation of VASP Ser157 and Ser239 could be induced by activation of the guanylyl cyclase C (GCC)/cGMP/protein kinase G (PKG) pathway; VASP Ser239 phosphorylation inhibits the biological behaviors of tumor cells. These findings show VASP phosphorylation at different sites to have varying regulatory effects on CC cells. Xiang *et al.* [54] suggested that knocking down VASP could inhibit the β1-integrin-focal adhesion kinase-1 (FAK)-yes-associated protein 1/tafazzin (YAP1/TAZ) signaling pathway, thereby inhibiting liver metastasis of gastrointestinal cancer. VASP has the ability to trigger extracellular matrix-mediated β1-integrin activation, which subsequently regulates the dephosphorylation of YAP1/TAZ downstream of RhoA. This modulation ultimately results in enhanced stability of the YAP1/TAZ protein. This indicates that VASP has great potential as a therapeutic target for CC and metastatic cancer. These studies have allowed us to understand the impact of VASP on CC and provided a more comprehensive understanding of the mechanism underlying the occurrence and development of CC.

### 4.6 VASP and Prostate Cancer

Li *et al.* [55] demonstrated that VASP is involved in regulating the invasive ability of PC3 cells, and that differences in VASP expression are associated with prostate cancer (PC) prognosis. Hasegawa *et al.* [56] showed that lysophosphatidic acid (LPA) mediates VASP phosphorylation at Ser157 *via* PKA to induce cellular lamellar pseudopod formation. Furthermore, the effect of LPA-induced lamellar pseudopod formation and cell migration was reduced by the knockdown of VASP or LPA receptor expression, suggesting that LPA receptor-induced phosphorylation of VASP is critical for migration initiation. Zhang *et al.* [57] demonstrated that microcystin-LR induced microfilament rearrangement and cell invasion of DU145 cells through the activation of the ERK/VASP/ezrin pathway. From the above studies, it can be seen that VASP has a regulatory effect on the aggressive ability of PC. However, in previous studies, there has been little research on the specific mechanisms, and the selected PC cell lines have been relatively single and error-prone. In future works, the relevant mechanisms of VASP regulation in PC cells should be studied, and various PC cell lines should be compared to reduce errors.

### 4.7 VASP and Other Types of Malignant Tumors

In addition to the abovementioned cancers, VASP has been studied in a small number of other types of malignancies. Zhao *et al.* [58] suggested that angiopoietin-like proteins regulate the activity of MMP- and EMT-related pathways by attenuating VASP phosphorylation, thereby inhibiting renal cell carcinoma metastasis. Nayak *et al.* [59] demonstrated that the level of phosphorylated VASP (Ser239) is upregulated by knocking down lysosome-associated membrane protein 3, thereby weakening the aggressiveness and metastasis of esophageal squamous cell carcinoma cells. Wu *et al.* [60] suggested that Rac1 knockdown leads to VASP expression in osteosarcoma cells, attenuating their migration and metastasis. Li *et al.* [61] showed that circRFX3 promoted glioma progression by regulating the miR-1179/miR-1229-VASP axis. Liu *et al.* [62] highlighted the important role of the bladder cancer-associated transcript 1 (BLACAT1)/miR-605-3p/VASP axis in promoting glioma cell invasion and migration. Tu *et al.* [63] showed that TGF-β stimulated hepatic stellate cells to differentiate into malignancies, but knocking down VASP could relieve this irritation. The above studies show that VASP plays a regulatory role in various tumor types. However, VASP has not been studied in some tumor types, and strengthening research on other tumors may provide new ideas for tumor development.

## 5 VASP IS INVOLVED IN THE REGULATION OF SIGNALING PATHWAYS IN TUMORS

**Fig. (3):**
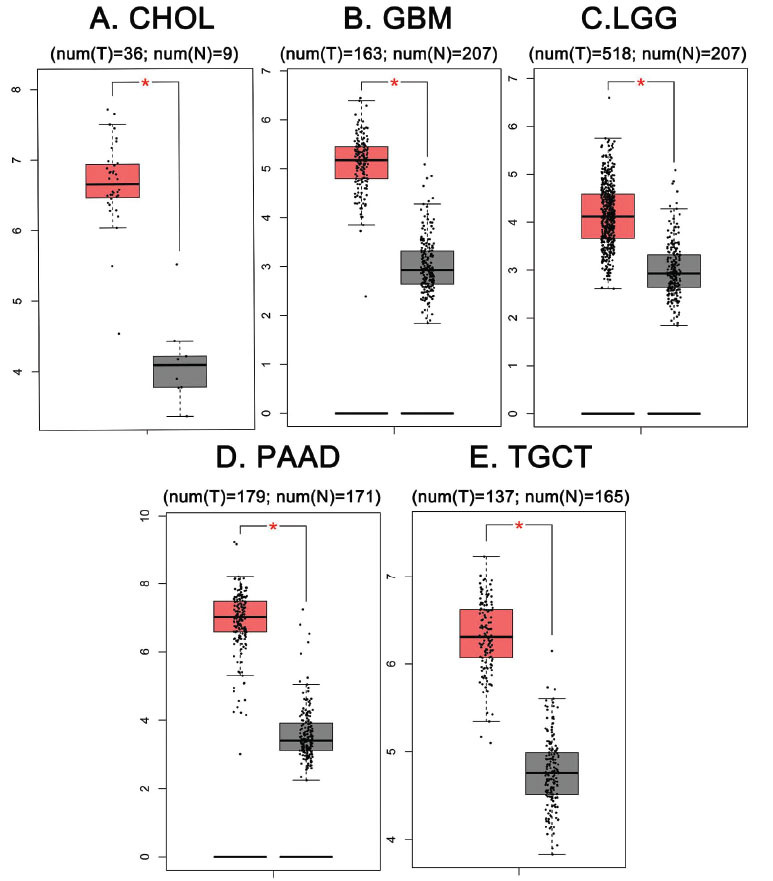
According to the GEPIA database, it was further found that the data of five tumor samples and paired normal tissues had significant differences in VASP expression. (**A**) The VASP gene expression of tumor samples was higher than paired normal tissues in CHOL (cholangiocarcinoma); the number of tumor samples was 36 and the number of paired normal tissues was 9 (num (T) = 26; num (N) = 9), *P* ≤ 0.05. (**B**) The VASP gene expression of tumor samples was higher than paired normal tissues in GBM (glioblastoma multiforme) (num (T) = 163, num (N) =207), *P* ≤ 0.05. (**C**) The VASP gene expression of tumor samples was higher than paired normal tissues in LGG (brain lower grade glioma) (num (T) =513, num (N) =207), *P* ≤ 0.05. (**D**) The VASP gene expression of tumor samples was higher than paired normal tissues in PAAD (pancreatic adenocarcinoma) (num (T) = 179; num (N) = 171), *P* ≤ 0.05. (**E**) The VASP gene expression of tumor samples was higher than paired normal tissues in TGCT (testicular germ cell tumors) (num (T) = 137, num (N) =165), *P* ≤ 0.05.

VASP is highly expressed in a variety of tumors and plays a key role in tumorigenesis (Fig. **3**); its overexpression is closely related to the activation and inactivation of multiple signaling pathways. Recent studies have demonstrated that VASP can participate in tumorigenesis, migration, and invasion through a variety of signaling pathways, thereby regulating biological functions, such as proliferation, apoptosis, invasion, and migration of tumor cells, which are closely related to tumor occurrence and development, clinical treatment, and patient prognosis; it also has broad research prospects in tumor diagnosis and prognosis biomarkers. Table **1** shows the signaling pathways in which VASP is involved in a variety of tumor cells.

## 6 INTERACTIONS OF PROTEINS RELATED TO VASP

**Fig. (4):**
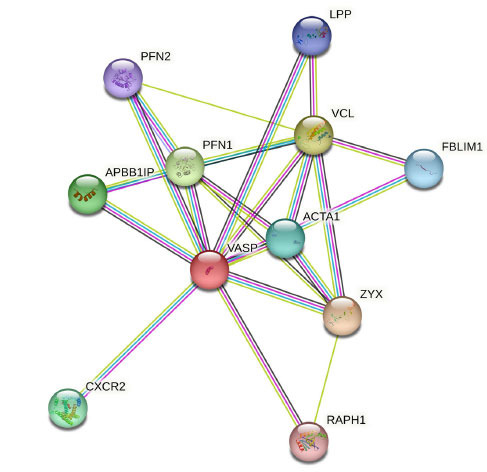
10 predicted interacting proteins associated with VASP from the String online website. Profilin-1 (PFN1), profilin-2 (PFN2), actin, alpha 1 skeletal muscle (ACTA1), filamin-binding LIM protein 1 (FBLIM1), vinculin (VCL), amyloid beta A4 precursor protein-binding family B member 1-interacting protein (APBB1IP), lipoma-preferred partner (LPP), zyxin (ZYX), C-X-C chemokine receptor type 2 (CXCR2), and Ras-associated and pleckstrin homology domains-containing protein 1 (RAPH1). Based on co-expression, experiments, databases, text mining, and homology for scoring, the four most likely interacting proteins were predicted: VCL, PFN1, ZYX, and APBB1IP (all scores being equal to 0.999).

**Fig. (5):**
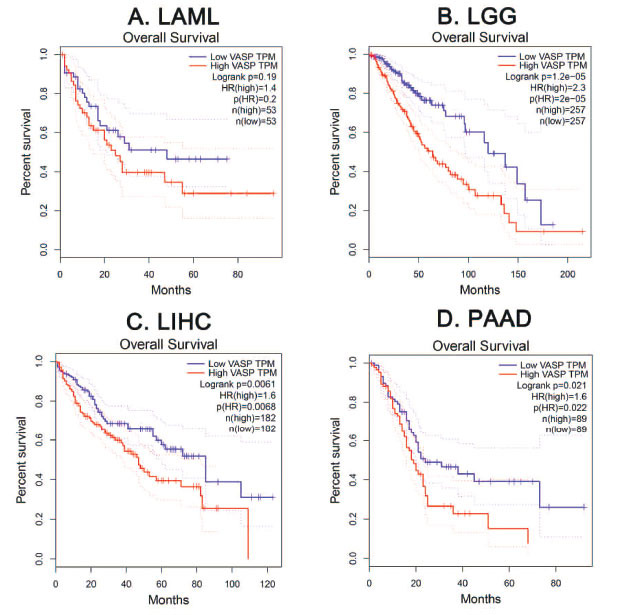
The effect of different expressions of VASP on overall survival in some patients with malignant tumors from the GEPIA database. Although there was no statistical difference, (**A**) the overall trend showed low VASP TPM to have a better over survival (OS) than high VASP TMP in LAML (acute myeloid leukemia); number high = 53, number low = 53 (n (high) = 26, n (low) = 9), (**B**) Low VASP TPM had a better OS than high VASP TMP in LGG (brain lower grade glioma) (n (high) = 257, n (low) =257), *P* ≤ 0.05, (**C**) Low VASP TPM had a better OS than high VASP TMP in LIHC (liver hepatocellular carcinoma) (n (high) = 182, n (low) =182), *P* ≤ 0.05, (**D**) Low VASP TPM had a better OS than high VASP TMP in PAAD (pancreatic adenocarcinoma) (n (high) = 182, n (low) = 182), *P* ≤ 0.05.

To predict genes that interact with VASP and understand their biological function, we searched for functional partners of VASP using the STRING (string-db.org) database (Fig. **4**). We searched the literature on interactions between VASP and functional partners according to the database results. The expression profiles of vinculin, VASP, and profilin proteins may indicate the adhesion and transformation status of epithelial cells, and they are coordinately regulated by signal transduction pathways affected by the translation response [64]. The zyxin-VASP complex is localized to cell-cell and cell-substrate adhesion sites, and regulates actin dynamics and actin-membrane junctions at these sites [65]. These studies illustrate the feasibility of this prediction model and provide ideas for future research (Fig. **5**).

## 7 DISCUSSION

Carcinoma is caused by abnormal cells that proliferate uncontrollably. The cytoskeleton plays a key role in determining the shape of cancer cells [25, 26]. Therefore, proteins that are functionally linked to actin are likely to play a role in promoting cancer cell invasion. Existing studies suggest that the invasion and metastasis of tumors are complex processes regulated by multiple steps and factors, but the migration ability of tumor cells is the key factor in determining tumor invasion and metastasis. Therefore, strengthening the regulation of the migratory ability of malignant tumor cells has important clinical significance for understanding and treating related diseases. Protrusion of lamellipodia is an important step in cell migration [66]. The formation of lamellipodia is based on the reconstruction of the cytoskeleton, which occurs during actin polymerization and depolymerization. As an important cytoskeletal regulatory protein, VASP, induced by lysophosphatidic acid, regulates tumor cell migration by affecting lamellipodia formation [56]. This suggests that VASP enhances tumor invasion and metastasis by promoting tumor cell migration.

Another biological feature of tumors is the formation of new blood vessels during tumorigenesis [67]. An *in vitro* experiment showed that VASP expression can promote capillary tube and network formation in a three-dimensional extracellular matrix [68]. Another study noted that vasculogenesis and endothelial sprouting during placental vasculogenesis may require VASP involvement [69]. Furthermore, the paracrine induction of VASP expression may be one of the roles of VEGF (vascular endothelial growth factor) and IL-8 (interleukin-8) in angiogenesis. In addition to its function in endothelial cells, VASP regulates the proliferation of muscle cells. VASP phosphorylation at Ser157 has a growth-stimulating effect, and VASP phosphorylation at Ser279 is critical for nitric oxide-dependent inhibition of vascular smooth muscle cell proliferation. Kim *et al.* [70] suggested that melanoma from VASP(-/-) has less angiogenesis compared to wild-type mice. In conclusion, VASP plays an important role in angiogenesis and has great potential as a therapeutic target for anti-angiogenesis in solid tumors.

Hypoxia represents a critical parameter of the tumor microenvironment in rapidly growing tumors, resulting from imbalanced oxygen consumption and insufficient vascular supply [71, 72]. The hypoxia microenvironment promotes multiple biological behaviors through the hypoxia-inducible factor (HIF) family [73, 74]. The hypoxia-dependent binding of HIF-1α to the human VASP promoter was demonstrated in a study that determined that HIF-1-dependent inhibition of VASP is the control point for hypoxia-regulatory barrier dysfunction [75]. In this review, we have also consulted the literature on the role of VASP-HIF-1α interactions in regulating tumor development. These studies illustrate that studying the interaction between VASP and HIF-1α is a promising direction in the field of tumor development.

In addition to studying the influence of VASP on tumorigenesis and development, the diagnostic value of VASP in various tumors is also a promising research direction. Immunohistochemistry (IHC) is an experimental technique that uses antigen-antibody reactions to determine antigens in tissue cells by making the color of the chromogen as that of labeled antibodies and localizing proteins. The proportion of positive expression of VASP in PC tissues was found to be increased, and strong positive expression of VASP tended to be associated with disease severity and survival prognosis [76]. Clinically, we can perform IHC on patients' tumor tissues to detect the staining intensity of VASP to guide the appropriate treatment plan and predict survival. Furthermore, using the GEPIA database (gepia.cancer-pku.cn), we found that VASP has varying degrees of impact on the overall survival of patients with various malignancies. This suggests that in future research, we can focus on the relationship between the expression of VASP and some clinical indicators. Therefore, some reasonable speculations regarding the direction of research can be drawn. High expression of VASP cloud be combined with some classic tumor markers (such as prostate-specific antigen, carcinoembryonic antigen, alpha-fetoprotein, *etc.*) to evaluate the prognosis of patients. After cancer patients receive chemoradiotherapy, it is also worth exploring to evaluate the treatment effect by detecting the changes in VASP expression in the blood.

In summary, VASP is closely linked to the occurrence and progression of malignant tumors. When VASP is inhibited, the invasive ability of tumor cells is significantly reduced. Moreover, strong positive expression of VASP can also predict faster biochemical recurrence. However, current research on VASP involves many deficiencies, such as the lack of research on the specific mechanism by which VASP affects the invasion and migration of some tumor cells, and the lack of survival analysis data of some clinical samples. Further research is required to confirm these findings. In follow-up studies, more attention should be paid to tumor regions where the role of VASP has not been explained in detail and more clinical samples should be collected to analyze the impact of different VASP expression levels on patient prognosis. There are many evident and promising directions for the study of VASP in future tumor research; however, VASP is involved in too many tumors and mechanisms. Among the many underlying mechanisms and diseases, our goal was to identify methods that are meaningful and clinically applicable.

## CONCLUSION

We have summarized the role of VASP in tumor development and progression according to the classification of malignant tumors. VASP is highly expressed in several malignant tumors; interacts with various molecules to promote cancer cell proliferation, migration, and invasion; and plays an important role as an oncogene during tumor development. However, there remains a gap in VASP research for some tumors, and there is great potential for further investigation.

## Figures and Tables

**Table 1:** VASP is involved in the regulation of signaling pathways in different tumors.

**Types of Tumors**	**The Regulation of VASP Expression on Tumorigenesis and Tumor Development**	**Pathway**	**Cell Line**	**References**
**Breast cancer**	Upregulation	Rac 1 TNF-α/HIF-1α/VASP CERB1/Lin28/miR-638/VASP Wnt/β-catenin PI3K/AKT	MDA-MB-231 MCF-7	[28] [29] [34] [35] [37]
**Gastric cancer**	Upregulation	Rac1 EGF/miR610/VASP PI3K/AKT NF-κB	BGC-823 MKN-28 SGC-7901	[41] [42] [43] [44]
**Lung cancer**	Upregulation	HIF-1α/VASP	A549	[47]
**Hepatocellular carcinoma**	Upregulation	AKT/ERK	MHCC-97H Hep3B SMMC-7721 Huh7 MHCC-97L HCCLM3	[48]
**Colon cancer**	Upregulation	GCC/cGMP/PKG β1-integrin-FAK-YAP1/TAZ	HEK 293T17 KM12L4 HCT116 HT29	[53] [54]
**Prostate cancer**	Upregulation	ERK/VASP/ezrin	DU145	[57]
**Osteosarcoma**	Upregulation	Rac1	Mg-63 Saos-2	[60]
**Glioma**	Upregulation	miR-1179/miR-1229-VASP BLACAT1/miR-605-3p/VASP	A172 U251 SHG44 LN299 U87 U251 LN18 U118 T98G	[61] [62]

## LIST OF ABBREVIATIONS

VASP: Vasodilator-stimulated Phosphoprotein
EVH1: Enable/VASP Homolog1
cAMP: 3',5'-Cyclic Adenosine Monophosphate
cGMP: 3′,5′-Cyclic Guanosine Monophosphate
PKA: Protein Kinase A
PKG: Protein Kinase G
PRR: Proline-rich
CNPs: Cluster-structured Nanoparticles
Mena: Mammalian Ena
PD-L1: Programmed Death-ligand 1
Arp: Actin-related Protein
GAB: G-actin Binding
BC: Breast Cancer
Rac1: Ras-related C3 Botulinum Toxin Substrate 1
TNF-α: Tumor Necrosis Factor-alpha
HIF-1α: Hypoxia-Inducible Factor-1alpha
EMT: Epithelial-mesenchymal Transition
PI3K: Phosphatidylinositol 3-kinase
AKT: Protein Kinase b
GC: Gastric Cancer
EGF: Epidermal Growth Factor
NF-κB: Nuclear Factor-kappa B
LC: Lung Cancer
HCC: Hepatocellular Carcinoma
ERK: Extracellular Signal-regulated Kinase
TGF-β: Transforming Growth Factor beta
MMPs: Matrix Metalloproteinases
IL-1β: Interleukin-1beta
CC: Colon Cancer
GCC: Guanylyl Cyclase C
PKG: Protein Kinase G
YAP1: Yes-associated Protein 1
TAZ: Tafazzin
PC: Prostate Cancer
IHC: Immunohistochemistry
